# Cats and external cervical resorption: Statistical considerations: Letter to editor for ‘Potential predisposing features of external cervical resorption: An observational study (Patel et al., 2025)’

**DOI:** 10.1111/iej.14258

**Published:** 2025-05-19

**Authors:** Nasir Z. Bashir, Eduardo Bernabé

**Affiliations:** ^1^ MRC Biostatistics Unit University of Cambridge Cambridge UK; ^2^ Institute of Dentistry Queen Mary University of London London UK

**Keywords:** cats, endodontics, epidemiology, external cervical resorption, statistics

## ETHICS STATEMENT

No ethical approval was required for this work.

Dear Editor,

We have read with some intrigue the recent article by Patel et al., 2025, which investigates putative risk factors for external cervical resorption (ECR). In a subsequent letter, Tay & Cooray, 2025, provide a fantastic exposition on why basic consideration of epidemiological methods means that drawing reliable inferences from this article is incredibly difficult. In addition to these concerns, we would like to highlight issues pertaining to the statistical methods used:

## POWER CALCULATIONS

The way in which the authors present their power calculations is an example of ‘cut‐and‐paste’ statistics (White et al., 2022). They claim ‘For a sample size of 180 patients, a power of 95% would be achieved to detect differences between two independent proportions, assuming a level of confidence of 95%’.

The formula for computing power with a binary outcome is dependent upon the proportion of events that occur in the two groups that are being compared. Assuming p_1_ and p_2_ are the proportions in groups 1 and 2 respectively, α and β are the type I and II error rates respectively, and Φ denotes the distribution function of the standard Normal, then to compute the sample size required in a two‐sample test for proportions:

$$n\approx \left(\left[p_{2}\left(1-p_{2}\right)+p_{1}\left(1-p_{1}\right)\right]/\left[p_{2}-p_{1}\right]\right){\left(\Phi \left[\beta \right]+\Phi \left[\alpha /2\right]\right)}^{2}$$

It is evident that power is dependent on what the investigator specifies p_1_ and p_2_ to be. The authors have not described what they assumed these to be, nor is there any mention of what the desired minimum detectable effect is, that is, p_2_–p_1_. Hence, it is completely unclear *what effect* the authors are claiming they have 95% power to detect.

For readers who wish to run the power calculation analogous to that which the authors present, we provide the following **R** code:**power.prop.test(n = 90**,**p1 = 0.5**,**p2 = NULL**,**sig.level = 0.05**,**power = 0.95**,**alternative = “two.sided”)**


The output of which indicates the authors have a power to detect a difference of 0.26 between proportions in independent groups, assuming p_1_ = 0.5. The differences in proportions between the groups observed in the study sample are generally far smaller than 0.26.

## MULTIPLE TESTING

The study sample was stratified into individuals with no identifiable factor, a single factor, or multiple factors. Whilst stratification in this manner is not inherently an issue, statistical tests carried out should be interpreted appropriately. In figure 4, there are 15 proposed risk factors. This means there are 2^15^ (= 31 768) possible combinations of risk factors which an individual may have. Taken to the logical extreme, should the authors not run all 31 768 tests and report all significant findings? Effectively, figures 6 through 8 suggest a less extreme version of this was carried out, with 15 tests per figure, resulting in (at a minimum) a total of 45 significance tests. Assuming a type I error rate of 0.05, this means that 2 to 3 of the reported significant findings are likely false positives. Tay & Cooray, 2025, suggest the use of correction for multiple testing to control the false discovery rate. There have been back‐and‐forth debates on the need to adjust for multiple testing for several decades, and we are not necessarily arguing that it should or should not have been done in this paper (Rothman, 1990). What we are stating, however, is that the issues around multiple testing should have been brought up and transparently discussed.

## STUDY DESIGN

The authors claim that ‘the aim of this study was to investigate potential predisposing factors associated with ECR’. All of the individuals in this study have ECR, so it is unclear exactly how this aim will be addressed, without comparison to individuals who do not have ECR.

This inappropriate study sample means that the null hypothesis in these significance tests which the authors carry out is not that the predisposing factors are associated with no increase in risk of ECR, that is, the intuitive null hypothesis which one typically assumes. Given that the study sample is patients with ECR, the findings of the statistical tests are to be interpreted as conditional on the presence of disease. For example, consider the reported result that ‘There was a significant association between cat ownership and ECR in the mandible (23.6%, 25/106 teeth, *p* = .002)’. This statement is not disingenuous, but it is still prone to misinterpretation by the reader. The null hypothesis being tested here is: amongst individuals with ECR, is cat ownership associated with jaw location? In other words, we are not being told if cat ownership is associated with an increased risk of ECR. We are being told, if you have ECR, is cat ownership associated with a difference in whether the ECR is incident in the upper or lower jaw? This hypothesis test seems effectively irrelevant to clinical practice.

More generally, the null hypotheses being across the statistical tests used were, ‘amongst individuals with ECR, is there an association between factor X and jaw location?’ Where factor X may be age, sex, cat ownership, number of putative risk factors etc. Again, it is completely unclear why such a test would be informative for clinical practice.

## CATS PROTECT AGAINST ECR?

Finally, and most obviously, the authors give only cursory consideration as to how cat ownership in their study sample relates to the population from which they are sampling. Of course, one should not forget (as Tay & Cooray, 2025, mention) naïve comparisons are prone to confounding and do not account for any causal structure. However, all the tests carried out in the article are done in this manner, so we feel equally justified in doing so.

The authors collected their data during the period from 2017 to 2022, and national statistics from Cats Protection indicate that the proportion of cat‐owning households in Greater London in 2021 was 26% (Cats Protection, 2021). Assuming the authors have taken a representative sample of patients with ECR, this means the prevalence of cat‐owning amongst patients with ECR is over 10% lower than that of the population from which they were sampled. Of course, this is a liberal estimate as one may need to correct for repeated measures. There were 194 patients and 215 teeth, so the upper bound on the proportion of cat‐owning ECR patients is 34/194 = 17.5% (95% CI: 12.2% to 22.9%); still much lower than the London population prevalence of cat‐owning households.

Based on the above data, should we then ask: does cat‐owning prevent ECR?

## AUTHOR CONTRIBUTIONS

Nasir Z. Bashir: Conceptualization, design, data acquisition and interpretation, drafting and critically revising the manuscript. Eduardo Bernabé: Drafting and critically revising the manuscript.

## FUNDING INFORMATION

NZB is supported by the Wellcome Trust (322 777/Z/24/Z).

## CONFLICT OF INTEREST STATEMENT

The authors declare no conflicts of interest.

## Data Availability

Data sharing not applicable to this article as no datasets were generated or analysed during the current study.

## References

[iej14258-bib-0001] Cats Protection . (2021) CATS Report 2021. Sussex, UK: Cats Protection. Available from: https://www.cats.org.uk/media/10005/cats‐2021‐full‐report.pdf [Accessed 9th April 2025].

[iej14258-bib-0002] Patel, S. , Abella, F. , Patel, K. , Bakhsh, A. , Lambrechts, P. & Al‐Nuaimi, N. (2025) Potential predisposing features of external cervical resorption: an observational study. International Endodontic Journal, 58(2), 273–283. 39560698 10.1111/iej.14166 PMC11715151

[iej14258-bib-0003] Rothman, K.J. (1990) No adjustments are needed for multiple comparisons. Epidemiology, 1(1), 43–46. 2081237

[iej14258-bib-0004] Tay, J.R.H. & Cooray, U. (2025) Letter to editor for “potential predisposing features of external cervical resorption: an observational study (Patel et al., 2025)”. International Endodontic Journal, 58(5), 797–798. 40200424 10.1111/iej.14215

[iej14258-bib-0005] White, N.M. , Balasubramaniam, T. , Nayak, R. & Barnett, A.G. (2022) An observational analysis of the trope “a p‐value of < 0.05 was considered statistically significant” and other cut‐and‐paste statistical methods. PLoS One, 17(3), e0264360. 35263374 10.1371/journal.pone.0264360 PMC8906599

